# Detecting network anomalies using Forman–Ricci curvature and a case study for human brain networks

**DOI:** 10.1038/s41598-021-87587-z

**Published:** 2021-04-14

**Authors:** Tanima Chatterjee, Réka Albert, Stuti Thapliyal, Nazanin Azarhooshang, Bhaskar DasGupta

**Affiliations:** 1 Department of Computer Science, University of Illinois at Chicago, Chicago, 60607 USA; 2 Department of Physics, Pennsylvania State University, University Park, 16802 USA

**Keywords:** Computer science, Complex networks

## Abstract

We analyze networks of functional correlations between brain regions to identify changes in their structure caused by Attention Deficit Hyperactivity Disorder (adhd). We express the task for finding changes as a network anomaly detection problem on temporal networks. We propose the use of a curvature measure based on the Forman–Ricci curvature, which expresses higher-order correlations among two connected nodes. Our theoretical result on comparing this Forman–Ricci curvature with another well-known notion of network curvature, namely the Ollivier–Ricci curvature, lends further justification to the assertions that these two notions of network curvatures are not well correlated and therefore one of these curvature measures cannot be used as an universal substitute for the other measure. Our experimental results indicate nine critical edges whose curvature differs dramatically in brains of adhd patients compared to healthy brains. The importance of these edges is supported by existing neuroscience evidence. We demonstrate that comparative analysis of curvature identifies changes that more traditional approaches, for example analysis of edge weights, would not be able to identify.

## Introduction

It is by now a common research practice to study the properties of complex interconnected systems by representing them as heterogeneous networks and then using various network-theoretic tools for their analysis^1,2^. Such heterogeneous networks may vary in diversity from simple undirected networks to edge-labeled directed networks. One such class of network models are *temporal* networks^3^ (networks whose edges vary over time) where *elementary components* of the network (such as nodes or edges) are added and/or removed as the network *evolves* over time. Examples of such networks include biological signal transduction networks with node dynamics, biochemical reaction networks, infectious disease contact networks, and time-evolving correlation networks^3^. Typically, such networks may have a set of *critical* elementary components (or simply “critical” components) whose presence or absence alters a significant *global* property of these networks between two time steps. Finding such a set of critical components in the context of temporal networks is more popularly called the *anomaly* detection or the *change-point* detection problem in statistics, computer science or data mining literature^4,5^, and prior widely used application areas of these problems include medical condition monitoring^6,7^, weather change detection^8,9^ and speech recognition^10,11^.

**Figure 1:**
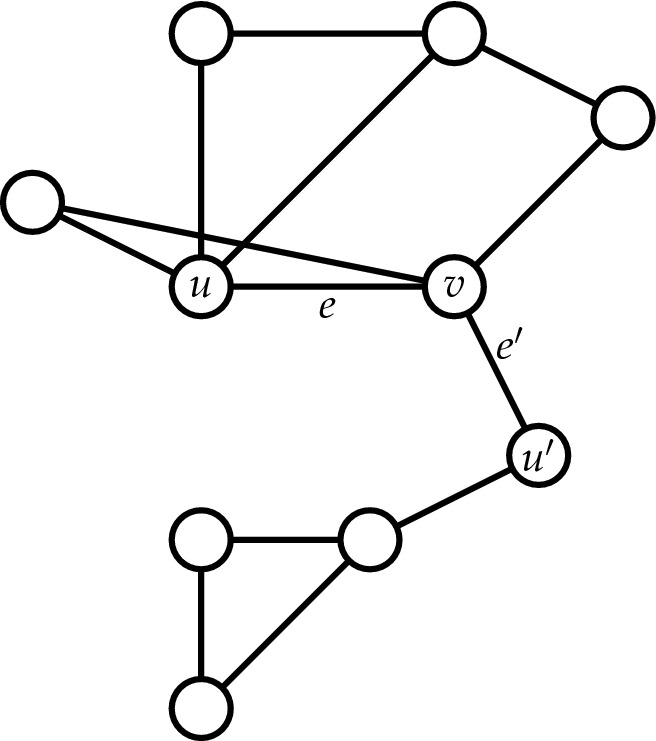
Illustration of a edge $e'$ that is a hanging edge (of order *d* for any *d*) with respect to the edge *e*.

**Figure 2:**
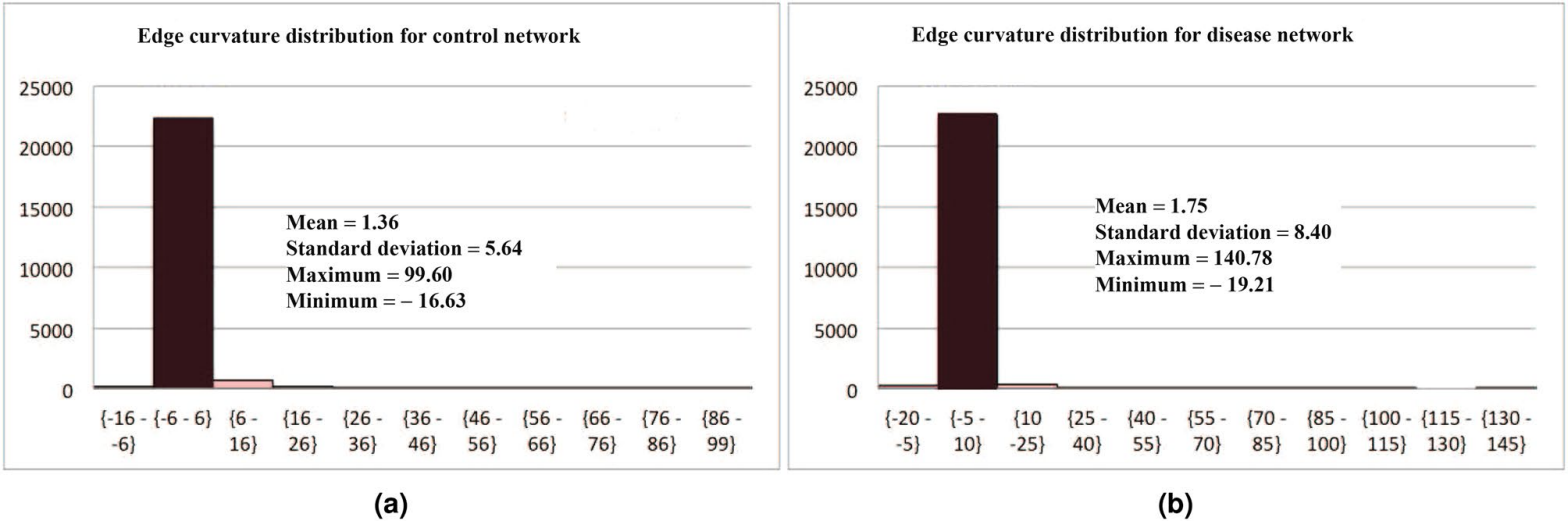
(**a**) Histogram showing the frequency of the edge curvatures in a given range for the control network. (**b**) Histogram showing the frequency of the edge curvatures in a given range for the disease network. For both (**a**) and (**b**), the heights of the bars along the *y*-axis indicate the number of samples belonging to the ranges given along the *x*-axis.

**Figure 3:**
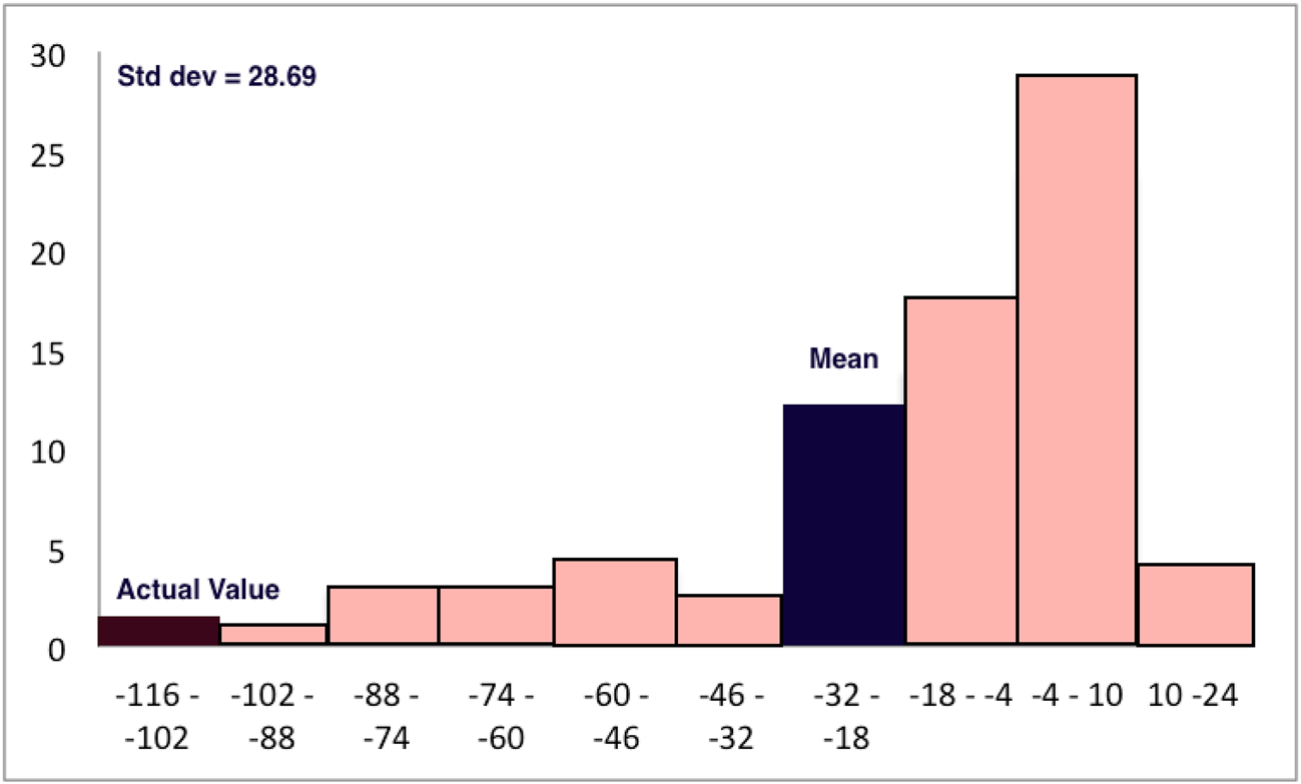
Histogram showing the frequency of the difference of edge curvatures among corresponding randomly generated network pairs of disease and control networks in a given range for the edge between L_Precuneous_Ctx_Superior_Lateral_Occipital_Ctx_128 and R_Superior_Lateral_Occipital_Ctx_Superior_Parietal_Lobule_Precuneous_Ctx_143 (the topmost edge in Table 2). The maroon bar indicates the range in which the curvature difference between the actual disease and control network belongs to while the blue bar indicates the population mean. The heights of the bars along the *y*-axis indicate the number of samples belonging to the ranges given along the *x*-axis.

**Figure 4:**
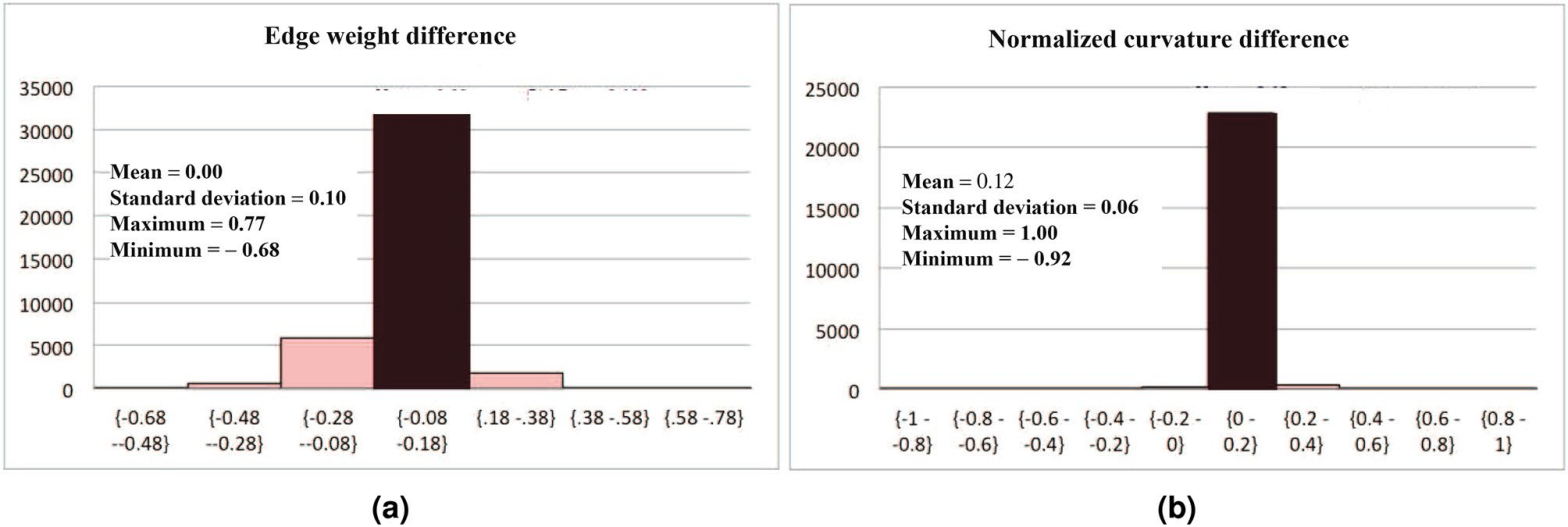
First-order statistics of the normalized curvatures differences and the edge weight differences over all pairs of nodes in the disease and the control network.

**Table 1:** The 32 outlier edges and their corresponding corresponding differences.

Edge name	Curvature difference $\pmb {\Delta _e}$
R_Superior_Parietal_Lobule_Lateral_Occipital_Ctx_12,	97.478
R_Superior_Lateral_Occipital_Ctx_Superior_Parietal_Lobule_Precuneous_Ctx_143	
L_Precuneous_Ctx_Superior_Lateral_Occipital_Ctx_128,	$-116.717$
R_Superior_Lateral_Occipital_Ctx_Superior_Parietal_Lobule_Precuneous_Ctx_143	
R_Insular_Ctx_Frontal_Oper_Ctx_3,	$-87.363$
L_Central_Opercular_Ctx_Frontal_Operculum_Ctx_Insular_Ctx_Putamen_198	
L_Frontal_Pole_1, L_Posterior_MTG_32	$-24.669$
L_Frontal_Pole_1, L_Frontal_Pole_47	$-62.764$
L_Frontal_Pole_1, L_Frontal_Pole_57	$-28.782$
L_Frontal_Pole_1, L_Superior_Lateral_Occipital_Ctx_Angular_Gyrus_68	$-18.273$
L_Frontal_Pole_1, L_Superior_Lateral_Occipital_Ctx_Agular_Gyrus_90	$-15.575$
L_Frontal_Pole_1, L_Frontal_Pole_FOC_92	$-29.940$
L_Frontal_Pole_1, R_SFG_120	$-16.636$
L_Frontal_Pole_1, L_Frontal_Pole_122	$-22.667$
L_Frontal_Pole_1, M_ACC_127	$-30.620$
L_Frontal_Pole_1, R_Frontal_pole_133	$-44.584$
L_Frontal_Pole_1, R_PCC_148	$-15.524$
L_Frontal_Pole_1, R_Frontal_Pole_197	42.766
R_Superior_Lateral_Occipital_Ctx_Precuneous_Ctx_20, L_Superior_Lateral_Occipital_Ctx_Precuneous_Ctx_98	45.556
R_Superior_Lateral_Occipital_Ctx_Precuneous_Ctx_20, L_Precuneous_Ctx_Superior_Lateral_Occipital_Ctx_128	$-48.615$
R_Occipital_Pole_Occipital_Fusiform_Gyrus_25, R_Inferior_Lateral_Occipital_Ctx_101	$-30.764$
L_Precentral_60, R_Postcentral_Superior_Parietal_Lobule_Precuneous_Ctx_158	$-54.354$
M_Juxtapositional_Lobule_Ctx_75,	$-105.182$
L_Central_Opercular_Ctx_Frontal_Operculum_Ctx_Insular_Ctx_Putamen_198	
R_Postcentral_Precentral_76, R_Postcentral_Superior_Parietal_Lobule_Precuneous_Ctx_158	$-28.090$
L_Postcentral_81, R_Postcentral_Superior_Parietal_Lobule_Precuneous_Ctx_158	$-70.806$
R_Insular_Ctx_Central_Opercular_Ctx_Putamen_89,	$-18.388$
L_Central_Opercular_Ctx_Frontal_Operculum_Ctx_Insular_Ctx_Putamen_198	
R_Planum_Polare_Anterior_STG_Temporal_Pole_Insular_Cortex_105,	17.111
R_Parietal_Operculum_Ctx_Planum_Temporale_Central_Operculum_Ctx_Heschls_193	
R_Planum_Polare_Anterior_STG_Temporal_Pole_Insular_Cortex_105,	$-17.616$
L_Central_Opercular_Ctx_Frontal_Operculum_Ctx_Insular_Ctx_Putamen_198	
L_Insular_Ctx_Central_Opercular_Ctx_Heschls_110,	$-23.146$
R_Parietal_Operculum_Ctx_Planum_Temporale_Central_Operculum_Ctx_Heschls_193	
R_SFG_120, R_Frontal_Pole_197	$-67.154$
L_Lingual_Gyrus_TOFFC_129, R_TOFFC_168	21.590
R_Posterior_STG_Planum_Temporale_Central_Opercular_Ctx_152,	55.522
R_Parietal_Operculum_Ctx_Planum_Temporale_Central_Operculum_Ctx_Heschls_193	
M_ACC_156, L_Central_Opercular_Ctx_Frontal_Operculum_Ctx_Insular_Ctx_Putamen_198	$-113.466$
L_Temporal_Pole_Anterior_ITG_163, R_Anterior_ITG_171	$-18.316$
R_TOFFC_168, R_Lingual_Gyrus_Occipital_Fusiform_Gyrus_179	22.628

The edge names are given in the format “u,v” where u and v are the names of the nodes in the network.

**Table 2:** 9 outlier edges with their curvature values and *z*-scores.

Edge name	Forman–Ricci curvature			
	Control	Diseased	Difference	$\pmb {z}$-score
	$\pmb {\mathfrak {C}^{\,2,5}_{G_1}(e)}$	$\pmb {\mathfrak {C}^{\,2,5}_{G_2}(e)}$	$\pmb {\Delta _e}$	of $\pmb {\Delta _e}$
** L_Precuneous_Ctx_Superior_Lateral_Occipital_Ctx_128,**	$\pmb {24.066}\,\,\,\,$	$\pmb {140.784}$	$\pmb {-116.717}$	$\pmb {3.3}$
** R_Superior_Lateral_Occipital_Ctx_Superior_Parietal_Lobule_Precuneous_Ctx_143**				
** M_ACC_156,**	$\pmb {1.050}\,\,\,\,$	$\pmb {114.517}$	$\pmb {-113.466}$	$\pmb {2.3}$
** L_Central_Opercular_Ctx_Frontal_Operculum_Ctx_Insular_Ctx_Putamen_198**				
** M_Juxtapositional_Lobule_Ctx_75,**	$\pmb {1.338}\,\,\,\,$	$\pmb {106.521}$	$\pmb {-105.182}$	$\pmb {2.3}$
** L_Central_Opercular_Ctx_Frontal_Operculum_Ctx_Insular_Ctx_Putamen_198**				
** R_Insular_Ctx_Frontal_Oper_Ctx_3,**	$\pmb {1.421}\,\,\,\,$	$\pmb {88.785}$	$\pmb {-87.363}$	$\pmb {2.5}$
** L_Central_Opercular_Ctx_Frontal_Operculum_Ctx_Insular_Ctx_Putamen_198**				
L_Postcentral_81, R_Postcentral_Superior_Parietal_Lobule_Precuneous_Ctx_158	$38.621\,\,\,\,$	109.428	$-70.806$	1.8
R_Frontal_Pole_1, L_Frontal_Pole_122	$10.066\,\,\,\,$	32.734	$-22.667$	1.4
R_Superior_Parietal_Lobule_Lateral_Occipital_Ctx_12,	$99.605\,\,\,\,$	2.126	97.478	$-1.3$
R_Superior_Lateral_Occipital_Ctx_Superior_Parietal_Lobule_Precuneous_Ctx_143				
R_Superior_Lateral_Occipital_Ctx_Precuneous_Ctx_20,	$47.386\,\,\,\,$	1.829	45.556	$-1.8$
L_Superior_Lateral_Occipital_Ctx_Precuneous_Ctx_98				
R_Superior_Lateral_Occipital_Ctx_Precuneous_Ctx_20,	$0.698\,\,\,\,$	49.314	$-48.615$	1.5
L_Precuneous_Ctx_Superior_Lateral_Occipital_Ctx_128				

The top 5 edges in the table are the top 5 of the 32 outlier edges, and the remaining 4 edges are selected from connecting those brain regions spanned by higher number of outlier edges. The edge names are given in the format “u,v” where u and v are the names of the nodes in the network. A *z*-score of absolute value at least 2 is generally considered to be statistically significant. Statistically significant curvature differences are shown in bold.

**Table 3:** The edge weight differences and the normalized curvature differences of the 9 outlier edges reported in Table 2 between the disease and the control network.

Edge name	Edge weight difference $\pmb {\Lambda _e}$	Normalized curvature difference $\pmb {\eta (\Delta _e)}$
L_Precuneous_Ctx_Superior_Lateral_Occipital_Ctx_128,	$-0.059$	$-0.927$
R_Superior_Lateral_Occipital_Ctx_Superior_Parietal_Lobule_Precuneous_Ctx_143		
M_ACC_156,	$-0.303$	$-0.898$
L_Central_Opercular_Ctx_Frontal_Operculum_Ctx_Insular_Ctx_Putamen_198		
M_Juxtapositional_Lobule_Ctx_75,	$-0.142$	$-0.823$
L_Central_Opercular_Ctx_Frontal_Operculum_Ctx_Insular_Ctx_Putamen_198		
R_Insular_Ctx_Frontal_Oper_Ctx_3,	$-0.194$	$-0.663$
L_Central_Opercular_Ctx_Frontal_Operculum_Ctx_Insular_Ctx_Putamen_198		
L_Postcentral_81, R_Postcentral_Superior_Parietal_Lobule_Precuneous_Ctx_158	$-0.106$	$-0.514$
R_Frontal_Pole_1, L_Frontal_Pole_122	$-0.017$	$-0.081$
R_Superior_Parietal_Lobule_Lateral_Occipital_Ctx_12,	$-0.081$	1.000
R_Superior_Lateral_Occipital_Ctx_Superior_Parietal_Lobule_Precuneous_Ctx_143		
R_Superior_Lateral_Occipital_Ctx_Precuneous_Ctx_20,	$-0.031$	0.533
L_Superior_Lateral_Occipital_Ctx_Precuneous_Ctx_98		
R_Superior_Lateral_Occipital_Ctx_Precuneous_Ctx_20,	$-0.107$	$-0.314$
L_Precuneous_Ctx_Superior_Lateral_Occipital_Ctx_128		

The edge names are given in the format “u,v” where u and v are the names of the nodes in the network.

In this paper, we will use the two terms “graph” and “network” *interchangeably*. We provide a formal definition of the network anomaly detection problem following a mathematical framework similar to what is described in^12^. To identify critical components of a temporal network, one first needs to provide details for the following four specific items:

(i): the network *model* under consideration,
(ii): a definition of the *elementary components* of the network, and
(iii): how the network *changes* over time,
(iv): the *property* of the network that will be used to identify *critical* components.

Let $t\ge 0$ be the time variable. For this paper, the details of these four items are as follows:

(i)’: The temporal network model considered in this paper is a *complete undirected* graph $G(t)=(V,E,\mathscr {w}(t))$ with a fixed set of *n* nodes *V*, a set of (all possible) edges $E=\{ \{u,v\} \,|\, u,v\in V,\,u\ne v \}$, and an edge-weight function $\mathscr {w}:E \times [0,\infty ) \mapsto {\mathbb R}^{+}\cup \{0\}$ that assigns a *non-negative real* number $\mathscr {w}(e,t)$ to each edge $e\in E$ for every time *t*. We will use the notations $\mathscr {w}(u,v,t)$, $\mathscr {w}(v,u,t)$ and $\mathscr {w}(e,t)$ interchangeably to indicate the weight of an edge $e=\{u,v\}$ at time *t*. Note that alternatively we could also view *G*(*t*) as a $n\times n$ connectivity matrix $A(t)=[a_{u,v}(t)]$ where the $(u,v)^{\mathrm {th}}$ entry of the matrix is given by $a_{u,v}(t)=\mathscr {w}(u,v,t)$.
(ii)’: The elementary components are the edges of the network, or equivalently following the convention as described above, they are every pair of nodes in the network.
(iii)’: The network changes over time by modification of these elementary components, i.e., the network changes over time by changing the weights of edges while keeping the *same* set of nodes. Note that a change of the weight of an edge from zero to a non-zero value is interpreted as the *addition* of a *new* edge, whereas a change of the weight of an edge from a non-zero value to zero is interpreted as the *deletion* of an *existing* edge.
(iv)’: The property of the network studied in this paper is the curvature of *G* given by a suitable version of the *Forman’s combinatorialization* of *Ricci curvature* for networks (henceforth will be referred to simply as the “*Forman–Ricci curvature*”)^13–17^. For now, assume that the Forman–Ricci curvature for a complete graph $G(t)=(V,E,\mathscr {w}(t))$ at time *t* is a real-valued scalar function $\mathfrak {C}_G : \big ( V\times V \setminus \{ (u,u) \,|\, u\in V\} \big ) \times [0,\infty ) \mapsto {\mathbb R}$ that maps every pair of nodes of *G* at time *t* to a real number with the assumption that
  - $\mathfrak {C}_G (u,v,t)= \mathfrak {C}_G (v,u,t)$, i.e., $\mathfrak {C}_G (u,v,t)$ is *symmetric* with respect to its first two arguments, and
  - $$\mathfrak {C}_G (u,v,t)=0$$ if $$\mathscr {w}(u,v,t)=0$$.

Then, our *network anomaly detection problem* can be defined as follows.

Given two complete graphs $G(t_1)=(V,E,\mathscr {w}(t_1))$ and $G(t_2)=(V,E,\mathscr {w}(t_2))$, find one or more pair(s) of nodes *u*, *v* such that the value of $|\mathfrak {C}_{G}(u,v,t_1)-\mathfrak {C}_{G}(u,v,t_2)|$ is sufficiently large.

In the above formulation of the network anomaly detection problem, we want $|\mathfrak {C}_{G}(u,v,t_1)-\mathfrak {C}_{G}(u,v,t_2)|$ to be sufficiently large since we want to identify large changes in the network to be more confident that the network is altered. This is equivalent to identifying connected components of the network with similar curvature values in the two time steps and focusing on the bridges among these components. Further technical details are provided later in the “Methods and materials” section. Here we solve the network anomaly detection problem in the context of changes of the human brain network caused by *Attention Deficit Hyperactivity Disorder* (adhd). adhd is one of the most common neuro-developmental disorders of childhood impacting parts of the brain that help us plan, focus on, and execute tasks. adhd impacts approximately 11% of children and 5% of adults in the US alone. It is usually first diagnosed in childhood and often lasts into adulthood. Children with adhd may have trouble paying attention, exhibit controlling impulsive behaviors, or be overly active. Unfortunately, the causes and risk factors for adhd are still unknown, and as of yet there is *no* single clinical test that helps diagnose adhd before its onset. There are several published neuroimaging studies that link the behavioral symptoms of adhd to altered connections between brain regions. In the last few years, network analysis methods have been extensively used for studying properties of human brain networks^18–21^. The human brain can be divided into different regions based on functional or anatomical properties^22^. One can consider these regions as nodes of a brain network and define the edges as functional correlations among brain regions. Two prior graph theoretical studies of adhd^23,24^ reported changes at the global level of the entire brain but did not study any altered connection patterns between different regions in the brain. In this paper we use our curvature-based network anomaly detection algorithms to detect statistically significant altered connection patterns between different regions of the brain.

Another related work^25^ characterized the brain networks affected by *Autism Spectrum Disorder* (asd) using *Ollivier–Ricci* curvature. Individuals with asd have altered white matter developmental patterns compared to individuals without asd. White matter development can be altered by neuro-inflammation, which in turn is associated with abnormalities in cerebrospinal fluid circulation. Autologous cord blood infusion, a potential therapy, is believed to reduce neuro-inflammation and promote white matter development, thus triggering a reconfiguration of connectivity patterns in the brain. Simhal et al. ^25^ used the Ollivier–Ricci network curvature (based on the mass transportation distance) to quantify the changes in the brain network after administering asd patients a single infusion of autologous umbilical cord blood. They calculated the Spearman correlation between changes in clinical behavioral scores and changes in curvature following treatment, and identified a relationship between clinical improvement and altered curvature in three white matter pathways that are implicated in social and communication abilities. We, on the other hand, use the combinatorial Forman–Ricci network curvature to determine and quantify the local and global changes in the brain network due to the onset of adhd and highlight the target nodes and edges which undergo major alterations due to the disease. We believe that our work in localizing the changes in the brain network will aid biomedical science in targeting specific treatment of the disease before the behavioral symptoms manifest.

### Notational simplifications

For simplified exposition and ensuring that notations are minimalistic, we will use the following conventions in suitable places through the article including supplementary documents S1.

- We may omit the time variable *t* from the argument of *G*, $\mathscr {w}$, $\mathfrak {C}_{G}$, or any other notation that uses *t* in its argument if it is completely clear from the context.
- We may interpret an edge *e* with $\mathscr {w}(e,t)=0$ as an edge that is actually *not* present in the given graph at time *t*.
- If we use the above conventions, then we will further simplify exposition by renaming the two (complete) input graphs $G(t_1)=(V,E,\mathscr {w}(t_1))$ and $G(t_2)=(V,E,\mathscr {w}(t_2))$ of the network anomaly detection problem as $G_1=(V,E_1,\mathscr {w}_1)$ and $G_2=(V,E_2,\mathscr {w}_2)$, respectively, where
  - $$E_1=E \setminus \{e \,|\, \mathscr {w}(e,t_1)=0\}$$,
  - $$E_2=E \setminus \{e \,|\, \mathscr {w}(e,t_2)=0\}$$,
  - $\mathscr {w}_1 : \{ (u,v) \,|\, u,v\in V,\,u\ne v \} \mapsto {\mathbb R}^{+}\cup \{0\}$ is given by $\mathscr {w}_1(u,v) = \mathscr {w}(u,v,t_1)$, and
  - $\mathscr {w}_2 : \{ (u,v) \,|\, u,v\in V,\,u\ne v \} \mapsto {\mathbb R}^{+}\cup \{0\}$ is given by $\mathscr {w}_2(u,v) = \mathscr {w}(u,v,t_2)$.

### Brief history of various notions of curvature for networks

Various notions of curvature are already widely used in disciplines such as physics and mathematics to study properties of high-dimensional objects of certain types ^26,27^. However, extensions of these curvature concepts to graphs and hyper-graphs are quite non-trivial for several reasons such as the discreteness and the lack of a preferred geometric embedding for combinatorial objects. There are several ways previous researchers have attempted to formulate such an extension in addition to what is used in this paper; we briefly review two such major approaches. We note that the references cited at various places in the paper also show that these kinds of network curvature measures can encode non-trivial topological properties that are not expressed by more established graph-theoretic measures such as degree distributions, clustering coefficients or betweenness centralities. Moreover, some of these references also show that these curvature measures can explain many phenomena one frequently encounters in real network-theoretic applications that are *not* easily explained by other measures.

The first kind of extension that gives rise to network curvatures involves some appropriate discretization of the Ricci curvature for a Riemannian manifold to capture metric properties of the manifold. Typically, these extensions defines a curvature value for every edge of a graph. In addition to the Forman–Ricci discretization used in this paper, another relevant and more direct discretization is the *Ollivier’s* discretization of Ricci curvature (the “Ollivier–Ricci” curvature)^28–31^. Roughly speaking, the Ollivier–Ricci curvature is calculated by defining a probability distribution on the neighborhoods of two nodes of an edge and then calculating the difference between the weight of the edge and the $L_1$ Wasserstein distance between the two above distributions. This kind of curvature is very different compared to the Forman–Ricci curvature used in this paper.

In another direction and historically much before the discretizations of Ricci curvature were formulated, Gromov and others defined a notion of network curvature (the ”Gromov-hyperbolic” curvature) via geodesic triangles that captures properties of the set of exact and approximate geodesics of the entire network^26,32^. There is a large body of research works dealing with various aspects of this measure, e.g., see^12,33–37^. The Gromov-hyperbolic curvature is a global measure in the sense that it assigns a scalar value to the entire graph, and therefore is not directly suitable for comparing components of two graphs.

There are also other notions of network curvatures explored in past literatures. For example, Chow and Luo^38^ provided a notion of network curvature based on circle packings.

## Methods and materials

### Formal definition of $\pmb {2}$-complex based Forman–Ricci network curvature

This paper used the combinatorial complex-based Forman–Ricci network curvature as defined below. For other notions of network curvatures, we refer the reader to references^26,28,30^. Assume that $G=(V,E,\mathscr {w})$ is the given graph with *n* nodes and *m* edges. For a formal definition we require some basic concepts from topology as available in introductory textbooks such as^39,40^; for convenience of the reader, we summarize these concepts in supplementary document S1. Conceptually, we define Forman–Ricci curvature of a network by “extrapolating” the network to higher-dimensional complexes via topological association^13^. To define such a topological association, the following definitions and assumptions are used for a simplicial complex:

- We define a partial order relation $\prec$ between faces of various dimensions of a simplex or a convex polytope in the usual manner: a $\ell$-face ${\mathfrak {f}}^{\,\ell }$ is a parent of a $\ell '$-face $\widehat{{\mathfrak {f}}}^{\,\ell '}$ (denoted by $\widehat{{\mathfrak {f}}}^{\,\ell '} \!\! \prec {\mathfrak {f}}^{\,\ell }$) if $\widehat{{\mathfrak {f}}}^{\,\ell '}$ is contained in ${\mathfrak {f}}^{\,\ell }$. Also, two $\ell$-faces ${\mathfrak {f}}^{\,\ell }$ and $\widehat{{\mathfrak {f}}}^{\,\ell }$ are *parallel* (denoted by ${\mathfrak {f}}^{\,\ell }\parallel \widehat{{\mathfrak {f}}}^{\,\ell }$) if they have either at least one common *immediate predecessor* or at least one common *immediate successor* (in the partial order $\prec$) *but not both*.
- We assume that there exists a weight function $\omega ({\mathfrak {f}})$ that assigns a non-negative weight (real number) to every face ${\mathfrak {f}}$. We will provide precise details of our implementation of the weight function in the next sub-section.

Informally, the higher-dimensional complex in the topological association is obtained by “gluing” nodes, edges, cycles and other sub-graphs of the given graph. There are many alternate ways such topological associations can be performed^12–14,41,42^. Our topological association is similar to that used in^12^ and is described as follows. For $q\in \{0,1,2\}$, we topologically associate a *q*-simplex with a $(q+1)$-clique $\mathscr {K}_{q+1}$, i.e., 0-simplexes, 1-simplexes and 2-simplexes are associated with nodes, edges and 3-cycles (triangles), respectively. Next, we use the concept of an *order* of a 2-simplex for more non-trivial topological association. Consider a *p*-face $f^p$ of a *q*-simplex for $q\in \{0,1,2\}$. An order *d* association of such a face, which we will denote by the notation $f_d^p$ with the additional subscript *d*, is associated with a sub-graph of *at most* *d* nodes that is obtained by starting with $\mathscr {K}_{p+1}$ and then *optionally* replacing each edge by a path between the two nodes. In other words,

- $f_d^0$ is a node of *G* for all $d\ge 1$.
- $f_2^1$ is an edge, and $f_d^1$ for $d>2$ is a path having at most *d* nodes between two nodes adjacent in *G*.
- $f_3^2$ is a triangle (cycle of 3 nodes or a 3-cycle), and $f_d^2$ for $d>3$ is obtained from 3 nodes in a 3-cycle by connecting every pair of nodes by a path such that the total number of nodes in the sub-graph is at most *d*.

For a node *v*, an edge *e* and a cycle $\mathscr {C}$, let the notations $v \sim e$ and $e \sim \mathscr {C}$ indicate that *v* is an end-point of *e* and *e* is an edge of $\mathscr {C}$, respectively. The basic formula of the 2-complex based order *d* Forman–Ricci curvature of an edge $e=\{u,v\}\in E$ is given by^13^:

1 $$\begin{aligned} \mathfrak {C}_G^{\,2,d}(e) {\mathop {=}\limits ^{\mathrm {def}}}\mathfrak {C}_{G}^{\,2,d}(u,v) = \omega (e) \left[ \left( \sum _{e \sim f_d^2} \frac{\omega (e)}{\omega (f_d^2)} \,{+}\, \sum _{v \sim e} \frac{\omega (v)}{\omega (e)} \right) \,\text {---}\, \sum _{e'||e} \left| \sum _{e',e \sim f_d^2} \frac{ \sqrt{\omega (e) \omega (e')} }{ \omega (f_d^2)} \,\text {---}\, \sum _{v\sim e,\,v\sim e'} \frac{ \omega (v)}{ \sqrt{\omega (e)\omega (e')} } \right| \,\,\, \right] \end{aligned}$$

Note that $\mathfrak {C}_{G}^{\,2,d}(u,v)=0$ if $\omega (e)=0$ as stated previously in the “Introduction” section.

Based on our need, we make one slight modification of the above formula. Finding all edges $e'$ such that $e'$ is parallel to an edge *e* would involve an worst-case running time of $O(n^d)$ which is quite prohibitive for us since we select *d* as 5. To make our calculations computationally tractable we therefore take only those edges parallel to *e* that belong to the same face, i.e., instead of $\sum _{e'||e}$ we use $\sum _{e'||e,\, e',e \sim f_d^2}$. Note that two parallel edges $e.e'$ belonging to the same face cannot have a common end-point. Thus, the simplified formula used in this paper is the following:

2 $$\begin{aligned} \mathfrak {C}_G^{\,2,d}(e) {\mathop {=}\limits ^{\mathrm {def}}}\mathfrak {C}_G^{\,2,d}(u,v) = \omega (e) \left[ \left( \sum _{e \sim f_d^2} \frac{\omega (e)}{\omega (f_d^2)} \,{+}\, \sum _{v \sim e} \frac{\omega (v)}{\omega (e)} \right) \,\text {---}\, \sum _{e'||e,\, e',e \sim f_d^2} \frac{ \sqrt{\omega (e) \omega (e')} }{ \omega (f_d^2)} \,\,\, \right] \end{aligned}$$

Equation (2) may look complicated at first glance to some readers. For the convenience of the reader, we illustrate the calculation of $\mathfrak {C}_G^{\,2,5}(e)$ for a small-size graph in supplementary document S2.

Let $\mathfrak {C}_G^{\,2,d}(e)^{(1)}$ and $\mathfrak {C}_G^{\,2,d}(e)^{(2)}$ be the values of $\mathfrak {C}_G^{\,2,d}(e)$ as computed by (1) and (2), respectively. What properties should a graph possess so that the two values $\mathfrak {C}_G^{\,2,d}(e)^{(1)}$ and $\mathfrak {C}_G^{\,2,d}(e)^{(2)}$ are identical? Call an edge $e'$ adjacent to the edge *e* if *e* and $e'$ share a node, i.e., if $e'$ is of the form $\{u,v'\}$ or $\{u',v\}$ for some $u'\ne u$ or $v'\ne v$. We define an edge $e'$ to be an *order d hanging edge* with respect to an edge $e=\{u,v\}$ (or, simply a hanging edge if other parameters are clear from the context) provided it satisfies the following two conditions (see Fig. 1 for an illustration):

1. The edges *e* and $e'$ are adjacent.
2. *G* does not contain a (simple) cycle of length (number of edges) at most *d* containing the edges *e* and $e'$.

#### Proposition 1

*If* *G* *has no hanging edges with respect to* *e* *then* $\mathfrak {C}_G^{\,2,d}(e)^{(1)}=\mathfrak {C}_G^{\,2,d}(e)^{(2)}$.

A proof of Proposition 1 is provided in supplementary document S3; note that the claim in Proposition 1 does *not* depend on the weights of the faces. Some examples of graph classes that satisfy the condition in Proposition 1 are as follows (these are just some examples, and do *not* necessarily list *every* possible graph class that satisfy the condition):

- The class of *complete* *k*-partite graphs for any $k\ge 3$ satisfy the condition in Proposition 1.
- The class of *complete* bipartite graphs with every partition having at least two nodes satisfy the condition in Proposition 1 for any $d\ge 4$.
- Any biconnected graph of circumference at most *d* satisfy the condition in Proposition 1.
- The class of standard hypercube graphs^43^ satisfy the condition in Proposition 1 for any $d\ge 4$. To see this, let $n=2^k$ and let $G=(V,E)$ be the standard *k*-dimensional hypercube where $V=\{(b_1,\dots ,b_k) \,|\, b_1,\dots ,b_k\in \{0,1\} \}$ and two nodes are connected by an edge if and only if they differ in exactly one coordinate. Let $u=(x_1,x_2,x_3,\dots ,x_k)$, $v=(1-x_1,x_2,x_3,\dots ,x_k)$, and $u'=(1-x_1,1-x_2,x_3,\dots ,x_k)$. Then, a cycle of length at most 4 containing the edges *e* and $e'$ consists of the edges $\{u,v\},\{v,u'\},\{u',u''\},\{u'',u\}$ where $u''=(x_1,1-x_2,x_3,\dots ,x_k)$.
- Increasing *d* allows more graph classes to satisfy the condition in Proposition 1. For the largest possible value of *d*, namely when $d=n$, all biconnected graphs satisfy the condition in Proposition 1.
- Consider the classical Erdös-Rényi random graph model^44^ *G*(*n*, *p*), namely the class of random graphs, parameterized by *p*, in which each possible edge $\{u,v\}$ is selected *independently* for inclusion in *G* with a probability of *p* for some $0<p<1$, and for convenience let the notation diam(*H*) denote the diameter of a graph *H*. The random graph $G(n,p)\setminus \{u,v\}$ obtained from *G*(*n*, *p*) by deleting the two nodes *u* and *v* and all the edges incident on these two nodes is itself an Erdös-Rényi random graph on $n-2$ nodes with the same *p* (since edges are selected independently). Note that *G*(*n*, *p*) satisfies the condition in Proposition 1 if $\text{ diam }(G(n,p)\setminus \{u,v\}) \le d-3$, and there are at most $$\left( {\begin{array}{c}n\\ 2\end{array}}\right) <n^2/2$$ choices of the two nodes *u* and *v*. Thus, using known extremal results for $\text{ diam }(G(n,p))$ and the union bound for probabilities we get the following bounds (the standard phrase “with high probability (*w.h.p.*)” in probabilistic methods refers to a probability of at least $1-o(1)$, i.e., a probability whose limit is 1 as *n* tends to infinity):
  - If $p\ge \frac{2 \log _2n}{n}$ then for all sufficiently large *n* *G*(*n*, *p*) satisfies the condition in Proposition 1*w.h.p.* for all $d\ge \frac{2 \log _2 n}{\log _2\log _2 n}$. For this result we need to use the bounds in Theorems 7.1 and 7.2 in^45^.
  - If *p* is a fixed constant less than 1 then for all sufficiently large *n* *G*(*n*, *p*) satisfies the condition in Proposition 1*w.h.p.* for all *d*. For this result we need to use the bounds in Corollary 10.11 in^46^.

Existence of a cycle $\mathscr {C}$ of at most *d* edges containing the edges *e* and $e'$ implies the existence of a path $\mathscr {P}$ between $u'$ and *v* of $\alpha \le d-2$ edges $\{u,u_1\}, \{u_1,u_2\}, \dots , \{u_{\alpha -1},u'\}$ where the nodes $u_1,\dots ,u_{\alpha -1}$ are distinct from the nodes $u'$ and *v*. As mentioned in the “Introduction” section, our graph could be viewed as an $n\times n$ connectivity matrix $A=[a_{u,v}]$ where zero entries correspond to edges *not* present in the graph. Viewed in this context, satisfying the condition in Proposition 1 is *tantamount* to satisfying the following claim:

for every ordered sequence of $\alpha -1\le d-3$ nodes from $V\setminus \{u,v,u'\}$, say the nodes $u_1,\dots ,u_{\alpha -1}$, at least one of the $\alpha$ quantities (henceforth to be referred to as the “weights of the sequence”) $\mathscr {w}(u,u_1), \mathscr {w}(u_1,u_2), \dots , \mathscr {w}(u_{\alpha -1},u')$ must be zero.

The total number $\Lambda$ of ordered sequences of at most $d-3$ nodes from $V\setminus \{u,v,u'\}$ is given by $$\Lambda = \sum _{j=1}^{d-3} \left( {\begin{array}{c}n-3\\ j\end{array}}\right) j ! $$. Since $\Lambda$ rapidly grows with *n* and *d*, it is unlikely that each of these $\Lambda$ ordered sequences will have at least one zero weight, unless *n* and *d* are relatively small or unless the connectivity matrix *A* has a *large* number of zeroes. For the connectivity matrix considered in this paper, $n=200$ and $d=5$, giving $$\Lambda =\left( {\begin{array}{c}197\\ 2\end{array}}\right) \times 2+\left( {\begin{array}{c}197\\ 1\end{array}}\right) =38809$$, a very large number.

### Details of the adhd brain networks analyzed in this study

The data for our empirical analysis was collected from the UCLA Multimodal Connectivity Database^47^ as available via the website http://umcd.humanconnectomeproject.org. This is a web based data repository with specific data analysis tools. The data here are in the form of connectivity matrices derived from neuroimaging data. The site is administered by MGH/UCLA Human Connectome Project. The source of the data available via various links in the website are of various imaging modalities such as fMRI, DTI, structural MRI and EEG, however the imaging modality for the specific study that we use is only fMRI. The data is also divided into various subject groups, ages, gender and spans different disorders such as Alzheimer’s, Autism, and adhd. Further details are available in supplementary document S5.

### Algorithmic and other specific details of our analysis of the adhd brain networks

Since computation of $\mathfrak {C}_G^{\,2,d}(e)$ via Eq. (2) requires (implicit or explicit) enumeration of cycles of up to *d* edges, the running time *is* prohibitive for arbitrary correlation matrices and arbitrarily large *d*. Thus, to reduce the time complexity we made the following decisions so that curvatures and *z*-scores can be computed within reasonable time:

- We selected $d=5$ so that using appropriate data structures we could still get the empirical results discussed in this paper within reasonable time.
- The original connectivity matrix had 200 nodes with a total of 34, 036 non-zero entries (edges). In order to reduce the computation time, we sparsified the given correlation matrix by zero-ing out all entries that are less than a threshold of 0.4. We observed that for a threshold of 0.3, the total number of non-zero entries was 27, 701 and our algorithm took a *very* long time to compute the edge curvatures. However, for a threshold of 0.4, the total number of non-zero entries was 25, 220 and the time taken was reduced to a *manageable* level. We also observed that for a higher threshold of 0.57 the graph corresponding to the non-zero entries in the thresholded connectivity matrix became disconnected, and the total number of non-zero edges was *significantly* reduced to 8800.

The computation of $\mathfrak {C}_G^{\,2,5}(e)$ requires a value for the weight $\omega (f_d^2)$ for $d\in \{4,5\}$. To satisfy this demand, we only considered those cycles that are “chordal”, i.e., they can be built by merging triangles along their common edge. We then specify the weight function $\omega$ for the given graph $G=(V,E,\mathscr {w})$ in the following manner:

- The weight $\omega (e)$ of an edge $e\in E$ is the correlation value of the edge, i.e., $\omega (e)=\mathscr {w}(e)$.
- The weight $\omega (v)$ of a node $v\in V$ is the average of the correlation values of the edges incident on *v*, i.e., $\omega (v)=\frac{ \sum _{v\sim e} \mathscr {w}(e) }{\deg (v)}$ where $\deg (v)$ is the degree of node *v*.
- The weight of a triangle (3-cycle) $f_3^2$ consisting of edges $e_1,e_2,e_3$ is $\omega (f_3^2) = (\mathscr {w}(e_1)+\mathscr {w}(e_2)+\mathscr {w}(e_3))/3$.
- The weight $\omega (f_d^2)$ for $d\in \{4,5\}$ were obtained by adding the weights of triangles from which $f_d^2$ was built.

### Evaluation of statistical significance

As briefly mentioned in the “Introduction” section, given the two graphs $G_1=(V_1,E_1,\mathscr {w}_1)$ and $G_2=(V_2,E_2,\mathscr {w}_2)$ and a pair of nodes *u*, *v* such that the *absolute value* of $\Delta _{u,v} = \mathfrak {C}^{\,2,5}_{G_1}(u,v)-\mathfrak {C}^{\,2,5}_{G_2}(u,v)$ is sufficiently large, we need to evaluate the statistical significance of this difference. For this purpose, we need a subroutine that, given a brain network $G=(V,E,\mathscr {w})$ and a pair of nodes $u,v\in V$, generates a random network $\mathscr {G}^{u,v}$ similar to *G* in the sense that the random network keeps the relation between the nodes *u* and *v* (i.e., if $\{u,v\}\in E$ then the edge is always kept but otherwise *u* and *v* are never connected by an edge), and preserves the first-order topological characteristics (such as the degree sequence) of *G* but randomizes higher-order statistics (such as distribution of paths) of *G*. Due to the absence of accurate generative null models for brain networks, we use the well-known and widely used Markov-chain algorithm for generating random networks^48^ that starts with the given network *G*, and repeatedly swaps randomly chosen pairs of connections up to $\eta$ times for a sufficiently large positive integer $\eta$ (for our specific implementation we selected $\eta =5|E|$). For the convenience of the reader, we provide the pseudo-code of the Markov-chain algorithm in supplementary document S4. This approach was used in papers such as^49,50^. Given such a subroutine for generating random networks, we use the following generic method which is by now standard in the network science literature dealing with various types of biological networks^49–51^. We repeat the following step 100 times: during the $j^{\mathrm {th}}$ iteration we generate two independent random networks $\mathscr {G}_{1,j}^{u,v}$ and $\mathscr {G}_{2,j}^{u,v}$ from $G_1$ and $G_2$, respectively, and calculate the value $\widetilde{\Delta }_{u,v,j} = \mathfrak {C}^{\,2,5}_{\mathscr {G}_{1,j}^{u,v} }(u,v)-\mathfrak {C}^{\,2,5}_{ \mathscr {G}_{2,j}^{u,v} }(u,v)$. We then calculate the standard *Z*-score^52^ of the observed value $\Delta _{u,v}$ with respect to the 100 samples $\widetilde{\Delta }_{u,v,1}, \widetilde{\Delta }_{u,v,2}, \dots , \widetilde{\Delta }_{u,v,100}$ as the desired statistical significance, and use a standard cut-off of 2 for labeling the *Z*-score as the criteria for being statistically significant. Using a *Z*-score to relate a property of an actual network to the distribution of the same property in an *ensemble* of randomized networks is a well established and popular method in network science, e.g., see^53^.

## Results

For our results, $G_1=(V,E_1,\mathscr {w}_1)$ and $G_2=(V,E_2,\mathscr {w}_2)$ correspond to the mean connectivity matrix of the 27 healthy individuals and the 24 diseased (adhd) patients, respectively, and henceforth will be referred to as the “*control network*” and the “*disease network*”, respectively. We implemented a program in PYTHON to efficiently compute $\mathfrak {C}^{\,2,5}$, and used it to compute the values of $\mathfrak {C}^{\,2,5}_{G_1}(u,v)$ and $\mathfrak {C}^{\,2,5}_{G_2}(u,v)$ for every pair of nodes *u* and *v*.

### Theoretical results

#### Comparison of Ollivier–Ricci curvature and Forman–Ricci curvature

References such as^28,54^ provide precise mathematical definitions of the Ollivier–Ricci curvature of a graph; for the convenience of the reader we do summarize these definitions in supplementary document S6. Let $\mathfrak {C}^{\mathrm {O}\text {-}\mathrm {R}}_{G}(e)$ denote the value of Ollivier–Ricci curvature of an edge *e* of a given graph $G=(V,E)$. It is not possible to directly compare the numerical values of $\mathfrak {C}^{\,2,d}_{G}(e)$ and $\mathfrak {C}^{\mathrm {O}\text {-}\mathrm {R}}_{G}(e)$ for a given graph *G* since $\mathfrak {C}^{\mathrm {O}\text {-}\mathrm {R}}_{G}(e)$ is a value in the range $[-2,1]$^54^ but $\mathfrak {C}^{\,2,d}_{G}(e)$ is a value in the range $[-\ell ,\ell ']$ for two numbers $\ell ,\ell '>0$ that depends on *n* and *m* via the topology of *G*. However, under the standard assumption that the zero value of a curvature represents the default flat curvature, one would expect the signs of the two curvatures to be *not* correlated if they indeed represent different discretization of the Ricci curvature for a Riemannian manifold. For further discussion, we recall the standard definition of the sign function ${{\,\mathrm{sgn}\,}}$:

$$\begin{aligned} {{\,\mathrm{sgn}\,}}(x)= \left\{ \begin{array}{ll} 1, &{} \text{ if } x>0 \\ \text{ undefined }, &{} \text{ if } x=0 \\ -1, &{} \text{ otherwise } \end{array} \right. \end{aligned}$$

The following theorem shows the lack of correspondence of the signs of the two curvatures.

##### Theorem 1

*For every* $s_1,s_2\in \{-1,1\}$ *and for every* $d\ge 5$ *the following claim is true: there exists an infinite family* $\mathscr {G}$ *of graphs such* *that for every graph* $G\in \mathscr {G}$ *it holds that* ${{\,\mathrm{sgn}\,}}(\mathfrak {C}^{\,2,d}_{G}(e))=s_1$ *and* ${{\,\mathrm{sgn}\,}}(\mathfrak {C}^{\mathrm {O}\text {-}\mathrm {R}}_{G}(e))=s_2$ *for* *some edge* *e* *of* *G*.

The proof of Theorem 1 is lengthy, requires review of some definitions and notations of prior literature, and uses Theorem 13.3 and its corollary in^55^; we therefore provide the proof in supplementary document S6. To the best of our knowledge, the result in Theorem 1 has not been formally proved before.

On a related note, Samal et al. ^17^ *empirically* compared $\mathfrak {C}^{\mathrm {O}\text {-}\mathrm {R}}_{G}(e))$ to some version of the Forman–Ricci curvature, and found that those two measures were correlated for a few real networks (similar assertions were also very briefly mentioned in^56^). However, as Samal et al. ^17^ themselves cautioned, their results should not be construed as implying that one of these curvature measures can be used as a *universal substitute* for the other measure, but merely that for some real networks using one of those measures that allow faster implementation may suffice. Our results in Theorem 1 lends further theoretical justification to their caution. There is *no* contradiction between our theoretical results and the empirical observations in^17,56^. To provide an analogy, consider the standard simplex algorithm for the linear programming problem. It has been theoretically proved that there exists examples of inputs for which for which simplex takes exponential time^57^. However, it is also a well-known observation that simplex runs in polynomial time for many empirical data^58^, and there is no contradiction between these two kinds of knowledge.

### Empirical results

#### First-order statistics of edge curvatures of control and disease networks

To begin, we display the first-order statistics of the curvatures of edges for the two networks in Fig. 2. These results shows that average curvature values of the two networks are *not* significantly different due to many edges of similar curvature values.

#### Edges with large statistically significant curvature differences

In accordance with the framework of our network anomaly detection problem as defined in the “Introduction” section, we next calculated the curvature differences $\Delta _{u,v} = \mathfrak {C}^{\,2,5}_{G_1}(u,v)-\mathfrak {C}^{\,2,5}_{G_2}(u,v)$ for every pair of nodes $u,v\in V$. The *average* and the *standard deviation* of the $\Delta _{u,v}$ values were found to be 0.384 and 6.9, respectively. Since the standard deviation was close to 7, we selected a value about twice the standard deviation (15) as a suitable cutoff for detecting drastic changes in curvature between corresponding edges. We found 32 pairs of nodes *u*, *v* which satisfied $|\Delta _{u,v}|>15$, and each such pair of node *u*, *v* correspond to an edge $e=\{u,v\}\in E_1\cap E_2$ (i.e., node pairs with non-zero correlation values) in *both* the control network $G_1$ and the disease network $G_2$ (and henceforth referred to as the 32 “*outlier edges*”). These edges and their corresponding curvature differences in the disease and control networks are shown in Table 1. Following are a few observations about these 32 outlier edges:

1. The top 2 outlier edges (i.e., the outlier edges with the largest and the second largest $|\Delta _{e}|$ values) belong to both the *occipital* region and to the *parietal lobe* region of the human brain.
2. 7 out of these 32 outlier edges lie in the *occipital* region of the brain.
3. 7 out of these 32 outlier edges belong to the *parietal lobe* region of the brain.
4. 12 out of these 32 outlier edges lie in the *left frontal pole* region of the brain.

The above findings show that most of the extreme curvature changes happen in the *occipital cortex* and the *frontal cortex* regions of the brain. This is consistent with previous neuroimaging studies of adhd involving voxel-wise estimation of regional tissue volume changes by Wang et al. ^59^. Their statistical results showed significant volume alteration in the brains of the patients with adhd. While there were significant volume *reductions* in the *prefrontal*, *parietal* and *temporal* regions, volume *enlargements* were observed in the *occipital regions* and *posterial lateral ventricle*. Along the same line, more recently Sun et al. in^60^ in 2017 conducted a comparative study by building a model using anatomic and diffusion-tensor MRI of different regions of the brains of children with adhd with that of children without the disease via MRI. They found that there were differences in the cortical shape of the frontal lobe and areas in the occipital lobe along with central cortex in the brains of adhd patients with those in (age and sex-matched) control groups.

We next focus our attention to the top 5 outlier edges (based on the $|\Delta _e|$ values) and 4 additional edges that connect brain regions spanned by a high number of outlier edges. In Table 2 we show these 9 outlier edges with their $\Delta _{e}$ values and, using the approach outlined in the “Methods and materials” section, the corresponding *z*-scores of the $\Delta _e$-values. Based on the standard assumption that a *z*-score of absolute value at least 2 is statistically significant, we note that 4 of the 9 outlier edges in Table 2 have statistically significant curvature differences. We make the following observations regarding these 4 edges with large and statistically significant curvature differences:

(a): The curvature values of all the four edges are higher in the disease network compared to the control network.
(b): One of the four edges, namely the topmost edge in Table 2, belong to the *occipital* region and the *superior parietal lobule* region of the brain.
  The *occipital* region of the brain is involved in *visual processing* activities such as object recognition, memory formation and distance perception, whereas the *superior parietal lobule* is important in planning movements, spatial reasoning and attention. As we have already mentioned before, the importance of these regions in adhd development is consistent with previous studies by Wang et al.^59^.
(c): The remaining three edges, namely the second, third and fourth edges from the top in Table 2, belong to the *occipital* region and the *frontal operculum* region of the brain.
  Generally speaking, the *frontal operculum* plays a role in thought, cognition, and planning behavior; for example, see the paper^61^ regarding the role of *operculum* in cognition and the subsequent changes in adhd patients. Studies such as^62^ suggest that the *frontal operculum* is a critical node in a brain network controlling activities in other brain areas to perform a wide array of cognitive tasks. Activities in the *frontal operculum* are involved in predicting the efficacy of future tasks, and also in cessation of engagement in a task. Decreased functional connectivities between the left frontal inferior operculum and other areas of brain were studied in^63^.

In addition, for illustrative purposes, we show in Fig. 3 the histogram of the frequency of the difference of edge curvatures among corresponding randomly generated network pairs of disease and control networks in a given range for the edge between L_Precuneous_Ctx_Superior_Lateral_Occipital_Ctx_128 and

R_Superior_Lateral_Occipital_Ctx_Superior_Parietal_Lobule_Precuneous_Ctx_143. As can be seen, the actual curvature difference of this edge *is* indeed an outlier with respect to the curvature differences of this edge in the random networks.

#### Edge curvature vs. edge weight for anomaly detection

A natural question that arises here is the following: “*why not simply use the edge-weights to find out significant differences between the disease and control network*?” Previous studies such as^64^ have indicated that edge weight by itself is not a sufficiently informative measure, for example the set of nodes with the highest sum of edge weights do not capture all the *hubs* in the brain networks. It is thus of interest to see the differences in edge weights between the corresponding edges in the two networks, and check if a measure as simple as the edge weight difference could also encode the higher order correlations that the curvature method encodes. Let us denote this difference of edge weights between the control and disease networks by $\Lambda _e$. We need to be careful to ensure a *fair* comparison between the $\Delta _e$ and the $\Lambda _e$ values since the $\Lambda _e$ values lie in the same range of $[-1,1]$, but this is *not* necessarily the case for the $\Delta _e$ values. Thus, for a fair comparison between the two sets of values, we “*normalize*” the $\Delta _e$ values to lie in the same range of $[-1,1]$ using the linear map $\eta : \Delta _e\mapsto \frac{2\Delta _e-\mathsf {high}-\mathsf {low}}{\mathsf {high}-\mathsf {low}}$ where $\mathsf {high}$ and $\mathsf {low}$ are the maximum and minimum, respectively, of $\Delta _e$ values over all edges *e*.

Figure 4 shows the first-order statistics of the normalized curvatures differences and the edge weight differences over all pairs of nodes in the disease and the control network. For both curvature differences and edge weight differences the standard deviations are very small, indicating that most individual values for them are concentrated within a small interval around their average values of 0.119 and 0, respectively. Table 3 shows the edge weight differences and the normalized curvature differences of the nine outlier edges reported in Table 2 between the diseased and control networks. As can be seen, the absolute values of all the actual edge weight differences are *significantly* smaller than the absolute values of their corresponding normalized curvature differences, and therefore edge weight differences would not have detected the same critical edges as our curvature differences.

## Discussion

Using the Forman–Ricci curvature measure as given by (2) in the framework of network anomaly detection, in this paper we have identified edges in brain networks of adhd patients versus healthy humans that exhibit statistically significant changes of the curvature measure. Our usage of the Forman–Ricci curvature measure focused on 2-complex based associations of order 5 with a specific scheme for computing weights of faces. The empirical analysis can be further improved by using *k*-complex based associations of order *d* for higher values of *k* and *d*. However, a practical limitation of the extent to which such refinements can be carried out in practice stems from the computational complexity issues. Another improvement may come from using different weighting scheme for the faces; for example, for our case Heron’s formula could not be used to provide weights of 3-cycles because it leads to complex numbers, but this obstacle may be avoided for other kinds of data.

Another direction for extension of our works in this paper would be to apply the Forman–Ricci curvature measure to analyze networks for other diseases such as schizophrenia, alzheimer’s disease or epilepsy. A further but equally important extension in this direction would be to use curvature measures to analyze the progression of a disease when suitable temporal data are available. For example, suppose that we have the brain network data over *k* time steps spanning the progress of a disease in a patient. This provides a series of networks $G^t$ for $t=1,\dots ,k$, and applying our analysis methods over successive pairs of networks $G^t,G^{t-1}$ for $t=2,\dots ,k$ may provide insights into the progression of interactions of various parts of the brain as the onset of the disease progresses in a patient. This type of analysis has been done by prior researchers using other more standard network measures, e.g., authors in^65,66^ showed that seizures evolve from a more random to regular and then back to random network structure before termination, but curvature analysis methods are likely to provide further non-trivial insights into the temporal structure of these networks.

A third direction of future research would be to compare another discretization of Ricci curvature, namely the Ollivier–Ricci curvature that was mentioned in Theorem 1, with the Forman–Ricci curvature in the context of our anomaly detection framework to compare the edges detected by the two measures. As we mentioned before, Samal et al. ^17^ did empirically compare the Ollivier–Ricci curvature to some version of the Forman–Ricci curvature for a few real networks, we believe a more comprehensive comparison may reveal salient distinguishing characteristics of these two curvatures for brain networks.

Finally, a further future research direction could be to investigate the biomedical significance of the curvature changes of the critical connections identified by curvature analysis methods such as ours.

## Supplementary Information

Supplementary Information

## Data Availability

The data for our empirical analysis is publicly available from the UCLA Multimodal Connectivity Database^47^ via the website http://umcd.humanconnectomeproject.org.

## Code availability

The source code for our program to compute $\mathfrak {C}_G^{\,2,5}(e)$ is freely available at https://github.com/Tchatt2/Brain/blob/main/5cycle.py.
