# Supplementary material for: Detecting network anomalies using Forman–Ricci curvature and a case study for human brain networks
Source: Sci Rep. 2021 Apr 14;11:8121. doi: 10.1038/s41598-021-87587-z (PMC8046810; doi:10.1038/s41598-021-87587-z)
Supplement: Supplementary file 1 — Supplementary Information [file 41598_2021_87587_MOESM1_ESM.pdf]

Supplementary documents

for

Detecting Network Anomalies Using Forman-Ricci Curvature and A Case Study for Human Brain Networks

Tanima Chatterjee, Réka Albert, Stuti Thapliyal, Nazanin Azarhooshang and Bhaskar DasGupta

Supplementary document S1

Summary of basic topological concepts

Here we review some basic concepts from topology; see introductory textbooks such as[39,40] for further information. For concreteness of exposition, let the underlying metric space be the r-dimensional real space $\mathbb{R}^r$ be for some integer $r > 1$.

- ▷ A subset $S \subseteq \mathbb{R}^r$ is *convex* if and only if for any pair $x, y \in S$, the *convex combination* of x and y is also in S (*i.e.*, $\lambda x + (1-\lambda)y \in S$ for any real $0 \leq \lambda \leq 1$).
- ▷ A set of $k+1$ points $x_0, \ldots, x_k \in \mathbb{R}^r$ are called *affinely independent* if and only if for all $\alpha_0, \ldots, \alpha_k \in \mathbb{R}$ $\sum_{j=0}^{k} \alpha_j x_j = 0$ and $\sum_{j=0}^{k} \alpha_j = 0$ implies $\alpha_0 = \cdots = \alpha_k = 0$.
- ▷ The *k-simplex* generated by a set of $k+1$ affinely independent points $x_0, \ldots, x_k \in \mathbb{R}^r$ is the subset of $\mathbb{R}^r$ $\mathcal{S}\big(x_0, \ldots, x_k\big) = \big\{ \sum_{j=0}^{k} \alpha_j x_j \,|\, \forall\, j : \alpha_j \geq 0 \text{ and } \sum_{j=0}^{k} \alpha_j = 1 \big\}$ generated by *all* convex combinations of $x_0, \ldots, x_k$. For example, the equation of a k-simplex with *unit intercepts* is given by $\sum_{j=0}^{k} x_j = 1$ with $x_j \geq 0$ for all $0 \leq j \leq k$.
  - ▷ Each $(\ell+1)$-subset $\big\{x_{i_0}, \ldots, x_{i_\ell}\big\} \subseteq \big\{x_0, \ldots, x_k\big\}$ defines the ℓ-simplex $\mathcal{S}\big(x_{i_0}, \ldots, x_{i_\ell}\big)$ that is called a *face* of dimension ℓ (or a ℓ-*face*) of $\mathcal{S}\big(x_0, \ldots, x_k\big)$. A $(k-1)$-face, 1-face and 0-face is called a *facet*, an *edge* and a *node*, respectively.
- ▷ A (closed) *halfspace* is a set of points satisfying $\sum_{j=1}^{r} a_j x_j \leq b$ for some $a_1, \ldots, a_r, b \in \mathbb{R}$. The convex set obtained by a bounded non-empty intersection of a finite number of halfspaces is called a *convex polytope* (called a *convex polygon* in two dimensions).
  - ▷ If the intersection of a halfspace and a convex polytope is a subset of the halfspace then it is called a *face* of the polytope. Of particular interests are faces of dimensions $r-1$, 1 and 0, which are called *facets*, *edges* and *nodes* of the polytope, respectively.
- ▷ A *simplicial complex* (or just a complex) is a topological space constructed by the union of simplexes.

Supplementary document S2

Illustration of calculation of $\mathfrak{C}_G^{2,5}(e)$ for a simple six-node network

Consider the 6-node graph shown in Figure S1, and assume that all edge weights are 1/2. We label various components of Equation (2) as follows:

$$\mathfrak{C}_G^{2,d}(e) = \omega(e)\left[\left(\underbrace{\sum_{e\sim f_d^2}\frac{\omega(e)}{\omega(f_d^2)}}_{\text{Term 1}} + \underbrace{\sum_{v\sim e}\frac{\omega(v)}{\omega(e)}}_{\text{Term 2}}\right) - \underbrace{\sum_{e'\|e,e',e\sim f_d^2}\frac{\sqrt{\omega(e)\omega(e')}}{\omega(f_d^2)}}_{\text{Term 3}}\right]$$

The various steps in the calculations for our graph $G=(V,E,\mathrm{w})$ are as follows (see Figure S1 for visual illustrations):

▷ The only f_3^2 containing the edge e is the 3-cycle $C_{2,3,4}$, the two f_4^2s containing the edge e are the 4-cycles $C_{2,3,4,5}$ and $C_{3,2,4,6}$, and the only f_5^2 containing the edge e is the 5-cycle $C_{3,2,5,4,6}$. The calculations of the relevant weight functions $\omega(\cdot)$ are as follows:

$$\omega(u_2) = \frac{\mathrm{w}(\{u_2,u_1\})+\mathrm{w}(\{u_2,u_3\})+\mathrm{w}(\{u_2,u_4\})+\mathrm{w}(\{u_2,u_5\})}{\deg(u_2)} = 1/2,\ \ \omega(u_3) = \frac{\mathrm{w}(\{u_3,u_2\})+\mathrm{w}(\{u_3,u_4\})+\mathrm{w}(\{u_3,u_6\})}{\deg(u_3)} = 1/2$$

$$\omega(e') = \mathrm{w}(e') = 1/2 \text{ for every edge } e' \in E$$

$$\omega(C_{2,3,4}) = \frac{\omega(e)+\omega(e_3)+\omega(e_4)}{3} = 1/2,\ \ \omega(C_{2,4,5}) = \frac{\omega(e_1)+\omega(e_2)+\omega(e_4)}{3} = 1/2,\ \ \omega(C_{3,4,6}) = \frac{\omega(e_3)+\omega(e_5)+\omega(e_6)}{3} = 1/2$$

$$\omega(C_{2,4,5}) = \frac{\omega(e_1)+\omega(e_2)+\omega(e_4)}{3} = 1/2$$

$$\omega(C_{2,3,4,5}) = \omega(C_{2,3,4})+\omega(C_{2,4,5}) = 1,\ \ \omega(C_{3,2,4,6}) = \omega(C_{2,3,4})+\omega(C_{3,4,6}) = 1$$

$$\omega(C_{3,2,5,4,6}) = \omega(C_{2,3,4})+\omega(C_{2,4,5})+\omega(C_{3,4,6}) = 3/2$$

▷ The total contributions of edge e to Term 1 and Term 2 are as follows:

$$\text{Contribution to Term 1 is } \alpha_1 = \frac{\omega(e)}{\omega(C_{2,3,4})} + \frac{\omega(e)}{\omega(C_{2,3,4,5})} + \frac{\omega(e)}{\omega(C_{3,2,4,6})} + \frac{\omega(e)}{\omega(C_{3,2,5,4,6})} = 7/3$$

$$\text{Contribution to Term 2 is } \alpha_2 = \frac{\omega(u_2)}{\omega(e)} + \frac{\omega(u_3)}{\omega(e)} = 2$$

▷ The calculations for Term 3 for various parallel edges are as follows:

 ▷ The edge e_2 is parallel to e in $C_{2,3,4,5}$ and $C_{3,2,5,4,6}$. The corresponding total contribution to Term 3 is as follows:

$$\gamma_1 = \frac{\sqrt{\omega(e)\omega(e_2)}}{\omega(C_{2,3,4,5})} + \frac{\sqrt{\omega(e)\omega(e_2)}}{\omega(C_{3,2,5,4,6})} = 5/6$$

 ▷ The edge e_5 is parallel to e in $C_{3,2,4,6}$ and $C_{3,2,5,4,6}$. The corresponding total contribution to Term 3 is as follows:

$$\gamma_2 = \frac{\sqrt{\omega(e)\omega(e_2)}}{\omega(C_{3,2,4,6})} + \frac{\sqrt{\omega(e)\omega(e_2)}}{\omega(C_{3,2,5,4,6})} = 5/6$$

▷ The final curvature value is then given by $\mathfrak{C}_G^{2,5}(e) = \omega(e)\times[\alpha_1+\alpha_2-\gamma_1-\gamma_2] = 4/3$.

Supplementary document S3

Proof of Proposition 1

Note that $\left| \sum_{(e' || e) \wedge (e', e \sim f_d^2)} \frac{\sqrt{\omega(e)\omega(e')}}{\omega(f_d^2)} \right|$ is the same as $\sum_{(e' || e) \wedge (e', e \sim f_d^2)} \frac{\sqrt{\omega(e)\omega(e')}}{\omega(f_d^2)}$ since all weights are positive numbers. Thus, it follows from a direct comparison of (1) and (2) that $\mathfrak{C}_G^{2,d}(e)^{(1)} = \mathfrak{C}_G^{2,d}(e)^{(2)}$ if the quantity $\Upsilon = \sum_{e' || e,\, v \sim e,\, v \sim e'} \frac{\omega(v)}{\sqrt{\omega(e)\omega(e')}}$ is zero. Obviously, Υ is zero if there is *no* such edge e' that satisfies the condition "$\Phi \equiv (e' || e) \wedge (v \sim e) \wedge (v \sim e')$" of the summation in the calculation of Υ. Φ is satisfied if and only if both the following conditions are satisfied:

(*a*) $(v \sim e) \wedge (v \sim e')$ is true, *i.e.*, edges e and e' are adjacent. Assume, without loss of generality, that $e' = \{u', v\}$ for the next condition.

(*b*) Edges e and e' are parallel edges. Since the edges e and e' have a common child (node) in the partial order $\prec$, the definition of parallel edges implies that e and e' must *not* have a common parent f_d^2 in the partial order $\prec$. Since f_d^2 in our topological association is a cycle of d edges, it follows that there is no cycle of length (number of edges) at most d in G containing the edges e and e'.

The proof is completed by noting that if G has no hanging edge that Φ is not satisfied since either **(*a*)** or **(*b*)** is not satisfied.

Supplementary document S4

Pseudo-code of the Markov-chain algorithm for generating random networks

repeat
  select a pair of distinct edges $e_1 = \{u_1, v_1\}, e_2 = \{u_2, v_2\} \in E \setminus \{u, v\}$ randomly uniformly
  if $\{u_1, v_1\} \cap \{u_2, v_2\} = \emptyset$ **then**
    if $\{u_1, v_2\} \notin E$ and $\{v_1, u_2\} \notin E$ **then**
      add the pair of edges $e'_1 = \{u_1, v_2\}$ and $e'_2 = \{v_1, u_2\}$ to E; set $w(e'_1) \leftarrow w(e_1)$ and $w(e'_2) \leftarrow w(e_2)$
      remove the pair of edges e_1 and e_2 from E
    else
      if $\{u_1, u_2\} \notin E$ and $\{v_1, v_2\} \notin E$ **then**
        add the pair of edges $e'_1 = \{u_1, u_2\}$ and $e'_2 = \{v_1, v_2\}$ to E; set $w(e'_1) \leftarrow w(e_1)$ and $w(e'_2) \leftarrow w(e_2)$
        remove the pair of edges e_1 and e_2 from E
  endif
until η pairs of edges have been swapped

Supplementary document S5

Details of data format collected from UCLA Multimodal Connectivity Database

The data that we used was in the form of two sets of mean/consensus matrices that are essentially the mean of the connectivity matrices of 27 healthy and 24 diseased patients respectively. The brain was categorized into 200 regions, hence each matrix had 200 nodes. Both the disease as well as control matrix had the *same* set of nodes. Each matrix comes in the form of the following text files:

- ▷ ADHD_grp_mean_24_region_names_abbrev_file.txt : This file essentially represents the abbreviations of the brain region names for the ADHD patients.
- ▷ ADHD_grp_mean_24_region_names_full_file.txt : This file contains the full names of the brain regions of the ADHD patients.
- ▷ ADHD_grp_mean_24_region_xyz_centers_file.txt : This file contains the (x,y,z) coordinates of the brain regions of the ADHD patients.
- ▷ ADHD_grp_mean_24_connectivity_matrix_file.txt : This file contains the actual mean connectivity matrix for the ADHD patients.
- ▷ CON_group_mean_27_region_names_abbrev_file.txt : This file contains the abbreviations of the brain region names for the control group.
- ▷ CON_group_mean_27_region_names_full_file.txt : This file contains the full names of the brain regions of the control group.
- ▷ CON_grp_mean_27_region_xyz_centers_file.txt : This file contains the (x,y,z) coordinates of the brain regions of the control group.
- ▷ CON_group_mean_27_connectivity_matrix_file.txt : This file contains the actual mean connectivity matrix for the control group.

Supplementary document S6

Proof of Theorem 1

We label various components of Equation (2) as follows:

$$\mathfrak{C}_G^{2,d}(e) = \omega(e)\left[\left(\underbrace{\sum_{e\sim f_d^2}\frac{\omega(e)}{\omega(f_d^2)}}_{\text{Term 1}} + \underbrace{\sum_{v\sim e}\frac{\omega(v)}{\omega(e)}}_{\text{Term 2}}\right) - \underbrace{\sum_{e'\|e,e',e\sim f_d^2}\frac{\sqrt{\omega(e)\omega(e')}}{\omega(f_d^2)}}_{\text{Term 3}}\right] \tag{3}$$

All of our constructions will satisfy the following claim: edge e' is parallel to e if and only if both e and e' belongs to some f_d^2. Thus, our proof will hold irrespective of whether we use (1) or (2) for the calculation of $\mathfrak{C}_G^{2,d}(e))$. For a fair comparison of the two curvatures using the definitions in[54] for the Ollivier-Ricci curvature, we will ensure that all edge weights in our constructions are 1, implying $\omega(v) = 1$ for all $v \in V$. For this case, we simplify (3) in the following manner. Let $\mathscr{F}_d^2 = \{f_d^2 \,|\, e \in f_d^2\}$ be the set of 2-faces of order d containing the edge e, and let $\eta_d^2 = |\mathscr{F}_d^2|$. Our constructions will ensure that all 2-faces of order d containing the edge e are triangulated for any $d > 3$ so that our weighting scheme described in the "Methods and Materials" section is applicable to these faces; in particular this implies that $\omega(f_d^2) = d-2$ for all $d \geq 3$. Term 2 in (3) evaluates to precisely 2 for our case. For any $d \geq 3$, a d-cycle $f_d^2 \in \mathscr{F}_d^2$ contributes $\frac{1}{\omega(f_d^2)} = \frac{1}{d-2} =$ to Term 1. Moreover, since such a f_d^2 has exactly $d-3$ edges parallel to e, it contributes $d-3/\omega(f_d^2)\frac{1}{d-2} = \frac{d-3}{d-2}$ to Term 3. Thus the total contribution over all d-cycles for any specific $d \geq 3$ is $\left(\frac{1}{d-2} - \frac{d-3}{d-2}\right)\eta_d^2 = \left(\frac{4-d}{d-2}\right)\eta_d^2$. Thus the curvature equation for our constructions simplify to the following:

$$\mathfrak{C}_G^{2,d}(e)) = 2 + \sum_{d\geq 3}\left(\frac{4-d}{d-2}\right)\eta_d^2 = 2 + \eta_3^2 - \sum_{d\geq 5}\left(\frac{d-4}{d-2}\right)\eta_d^2 \tag{4}$$

Next we briefly review key definitions and notations from[54] as needed in our proof of Theorem 1; for further details see the original paper[54]. Let $G = (V,E)$ be a given undirected unweighted graph. The following two notations are used subsequently:

- ▷ $\mathsf{Nbr}(v) = \{u \,|\, \{v,u\} \in E\}$ and $\deg(v) = |\mathsf{Nbr}(v)|$ are the set of neighbors and the degree, respectively, of a node $v \in V$.
- ▷ $\text{dist}_G(u,v)$ is the *distance* (*i.e.*, number of edges in a shortest path) between the nodes u and v in G.

Fix an edge $\{u,v\} \in E$. Let $V_{u,v} = \{u,v\} \cup \mathsf{Nbr}(u) \cup \mathsf{Nbr}(v)$, and define two probability distributions $\mathbb{P}_u$ and $\mathbb{P}_v$ over the nodes in $V_{u,v} = \{u,v\}$ as follows:

$$\mathbb{P}_u(x) = \begin{cases} \frac{1}{1+\deg(u)}, & \text{if } x \in \{u\} \cup \mathsf{Nbr}(u) \\ 0, & \text{otherwise} \end{cases} \qquad \mathbb{P}_v(x) = \begin{cases} \frac{1}{1+\deg(v)}, & \text{if } x \in \{v\} \cup \mathsf{Nbr}(v) \\ 0, & \text{otherwise} \end{cases}$$

The *Earth Mover's Distance* corresponding to these two probability distributions, denoted by $\text{EMD}(V_{u,v},\mathbb{P}_u,\mathbb{P}_v)$, is the value of the objective function of an optimal solution of the following linear program with a variable $z_{u,v}$ for every pair of nodes $u,v \in V_{u,v}$:

$$\begin{aligned} &\textit{minimize} \quad \textstyle\sum_{u\in V_{u,v}}\sum_{v\in V_{u,v}} \text{dist}_G(u,v)\, z_{u,v} \\ &\textit{subject to} \\ &\quad \textstyle\sum_{v\in V_{u,v}} z_{u',v} = \mathbb{P}_u(u'), \text{ for each } u' \in V_{u,v} \\ &\quad \textstyle\sum_{u\in V_{u,v}} z_{u,v'} = \mathbb{P}_v(v'), \text{ for each } v' \in V_{u,v} \\ &\quad z_{u,v} \geq 0, \text{ for all } u,v \in V_{u,v} \end{aligned}$$

The Ollivier-Ricci curvature is then defined as $\mathfrak{C}_G^{\text{O-R}}(e) = 1 - \text{EMD}(V_{u,v},\mathbb{P}_u,\mathbb{P}_v)$. For calculation of $\mathfrak{C}_G^{\text{O-R}}(e)$ for our constructions, it will be useful to prove the following result.

Lemma 1. *Fix* $\gamma \in \{1,2,3\}$. *Consider an edge* $e = \{u,v\}$ *of the graph* $G = (V,E)$ *such that* $\deg(u) = \deg(v) = \alpha > 1$ *and* $\text{dist}_G(u',v') = \gamma$ *for every* $u' \in \mathsf{Nbr}(u) \setminus (\mathsf{Nbr}(v) \cup \{v\})$ *and* $v' \in \mathsf{Nbr}(v) \setminus (\mathsf{Nbr}(u) \cup \{u\})$. *Then, letting* $\beta = |\mathsf{Nbr}(u) \setminus (\mathsf{Nbr}(v) \cup \{v\})| = |v' \in \mathsf{Nbr}(v) \setminus (\mathsf{Nbr}(u) \cup \{u\})|$ *it follows that*

$$\text{EMD}(V_{u,v},\mathbb{P}_u,\mathbb{P}_v) = \frac{\gamma\beta}{\alpha+1} \quad \equiv \quad \mathfrak{C}_G^{\text{O-R}}(e) = 1 - \text{EMD}(V_{u,v},\mathbb{P}_u,\mathbb{P}_v) = 1 - \frac{\gamma\beta}{\alpha+1}$$

Proof. Consider the edge-weighted complete bipartite graph $H=(A,B,F,w)$, where there is a node $a_{u'}\in A$ for every node $u'\in \mathsf{Nbr}(u)\cup\{u\}$, there is a node $b_{v'}\in A$ for every node $v'\in \mathsf{Nbr}(v)\cup\{v\}$, and the weight function $w: A\times B\mapsto\{0,1,2,3\}$ is given by $w(u',v')=\mathrm{dist}_G(u',v')$. Build a directed single-source single-sink flow network[55] $H_f=$ from H in the following manner: add a new source node s and a new sink node t, add an arc (directed edge) from s to every node of A of weight zero and capacity 1, add an arc from every node of B to t of weight zero and capacity 1, orient every edge $\{a_{u'},b_{v'}\}\in F$ from $a_{u'}$ to $b_{v'}$ and set its capacity to 1. Since $|A|=|B|=\alpha+1$, we have $\mathbb{P}_u(u')=\mathbb{P}_v(v')=\frac{1}{\alpha+1}$ for all $u'\in \mathsf{Nbr}(u)\cup\{u\}$ and $v'\in \mathsf{Nbr}(v)\cup\{v\}$. Thus, since H is a complete bipartite graph, by a simple scaling it follows that $\textsc{Emd}(V_{u,v},\mathbb{P}_u,\mathbb{P}_v)=\frac{\mathscr{M}}{\alpha+1}$ where $\mathscr{M}$ is the total weight of a minimum-weight maximum s-t flow on H_f. Since the node-arc incidence matrix of a directed graph is totally unimodular, the flow value of every arc of any extreme-point optimal solution of the minimum-weight maximum s-t flow on H_f is integral and therefore 0 or 1 (see Theorem 13.3 and its corollary in[55]). This integrality of flow values and the fact that H is a complete bipartite graph imply $\mathscr{M}$ is also the total weight of a minimum-weight *perfect* matching of H.

We now estimate the total weight $\mathscr{M}$ of a minimum-weight *perfect* matching of H in the following manner. Let $A'=\{a_{u'}\,|\,u'\in\{u,v\}\cup(\mathsf{Nbr}(u)\cap\mathsf{Nbr}(v))\}$ and $B'=\{b_{v'}\,|\,v'\in\{u,v\}\cup(\mathsf{Nbr}(u)\cap\mathsf{Nbr}(v))\}$. Note that the edge-weights of H satisfy the following based on our assumptions: $\mathrm{dist}_G(a_{u'},b_{v'})=0$ if $a_{u'}\in A'$, $b_{v'}\in B'$, $u'=v'$. We claim that there exists a minimum-weight perfect matching of H that uses the edges $\{a_{u'},b_{u'}\}$ for all $u'\in\{u,v\}\cup(\mathsf{Nbr}(u)\cap\mathsf{Nbr}(v))\}$. For a contradiction, suppose that the edge $\{a_{u'},b_{u'}\}$ is not used for some $u'\in\{u,v\}\cup(\mathsf{Nbr}(u)\cap\mathsf{Nbr}(v))\}$. Since our solution is a perfect matching, the nodes $a_{u'}$ and $b_{u'}$ must be matched to some other nodes, say to nodes $b_{v''}\in B$ and $a_{u''}\in A$, respectively. Then, if we instead use the edges $\{a_{u'},b_{u'}\}$ and $\{a_{u''},b_{v''}\}$ then using the total weight of this modified perfect matching is no more than that of the original perfect matching since

$$w(a_{u'},b_{u'})+w(a_{u''},b_{v''})=w(a_{u''},b_{v''})\le w(a_{u''},b_{u'})+w(a_{u'},b_{u'})+w(a_{u'},b_{v''})=w(a_{u''},b_{u'})+w(a_{u'},b_{v''})$$

Thus, there exists a perfect matching that $\{a_{u'},b_{u'}\}$ for all $u'\in\{u,v\}\cup(\mathsf{Nbr}(u)\cap\mathsf{Nbr}(v))\}$ of total weight 0, and some perfect matching of the nodes in $A\setminus A'$ and $B\setminus B'$. Since $\mathrm{dist}_G(a_{u'},b_{v'})=\gamma$ for all $a_{u'}\in A\setminus A'$ and $b_{v'}\in B\setminus B'$, we get $\mathscr{M}=\gamma\times|A\setminus A'|=\gamma\beta$, and the desired bound follows. □

We now continue with the proof of Theorem 1.

Proof for $s_1=1, s_2=1$

Consider the n-node graph as shown in Figure S2a where $n>2$. Since $\eta_3^2=1$ and $\eta_d^2=0$ for all $d\ge5$, using (4) we get $\mathfrak{C}_G^{2,d}(e))=2+\eta_3^2=3>0$. For the Ollivier-Ricci curvature, note that (in the notations of Lemma 1 and[54]) $\beta=0$ giving $\mathfrak{C}_G^{\text{O-R}}(e)=1>0$.

Proof for $s_1=1, s_2=-1$

Consider the n-node graph as shown in Figure S2b where $n>25$. By straightforward calculation $\eta_3^2=6$, $\eta_5^2=15$, $\eta_d^2=0$ for all $d>5$, and thus using (4) we get $\mathfrak{C}_G^{2,d}(e))=2+\eta_3^2-\frac{\eta_5^2}{3}=3>0$. For the Ollivier-Ricci curvature, note that (in the notations of Lemma 1 and[54]) $\alpha=16$, $\beta=9$ and $\gamma=2$, giving $\mathfrak{C}_G^{\text{O-R}}(e)=1-\frac{2\times9}{17}=-\frac{1}{17}<0$.

Proof for $s_1=-1, s_2=1$

Consider the n-node graph as shown in Figure S2c where $n>5$. By straightforward calculation $\eta_3^2=4$, $\eta_5^2=\eta_6^2=24$, $\eta_d^2=0$ for all $d>6$, and thus using (4) we get $\mathfrak{C}_G^{2,d}(e))=2+\eta_3^2-\frac{\eta_5^2}{3}-\frac{\eta_6^2}{2}=-14<0$. For the Ollivier-Ricci curvature, note that (in the notations of Lemma 1 and[54]) $\beta=0$ giving $\mathfrak{C}_G^{\text{O-R}}(e)=1>0$.

Proof for $s_1=-1, s_2=-1$

Consider the n-node graph as shown in Figure S2d where $n>10$. By straightforward calculation $\eta_3^2=1$, $\eta_5^2=16$, $\eta_d^2=0$ for all $d>5$, and thus using (4) we get $\mathfrak{C}_G^{2,d}(e))=2+\eta_3^2-\frac{\eta_5^2}{3}=-\frac{7}{3}<0$. For the Ollivier-Ricci curvature, note that (in the notations of[54]) $\alpha=6$, $\beta=4$ and $\gamma=2$, giving $\mathfrak{C}_G^{\text{O-R}}(e)=1-\frac{2\times4}{7}=-\frac{1}{7}<0$.

Supplementary figures

for

Detecting Network Anomalies Using Forman-Ricci Curvature and A Case Study for Human Brain Networks

Tanima Chatterjee, Réka Albert, Stuti Thapliyal, Nazanin Azarhooshang and Bhaskar DasGupta

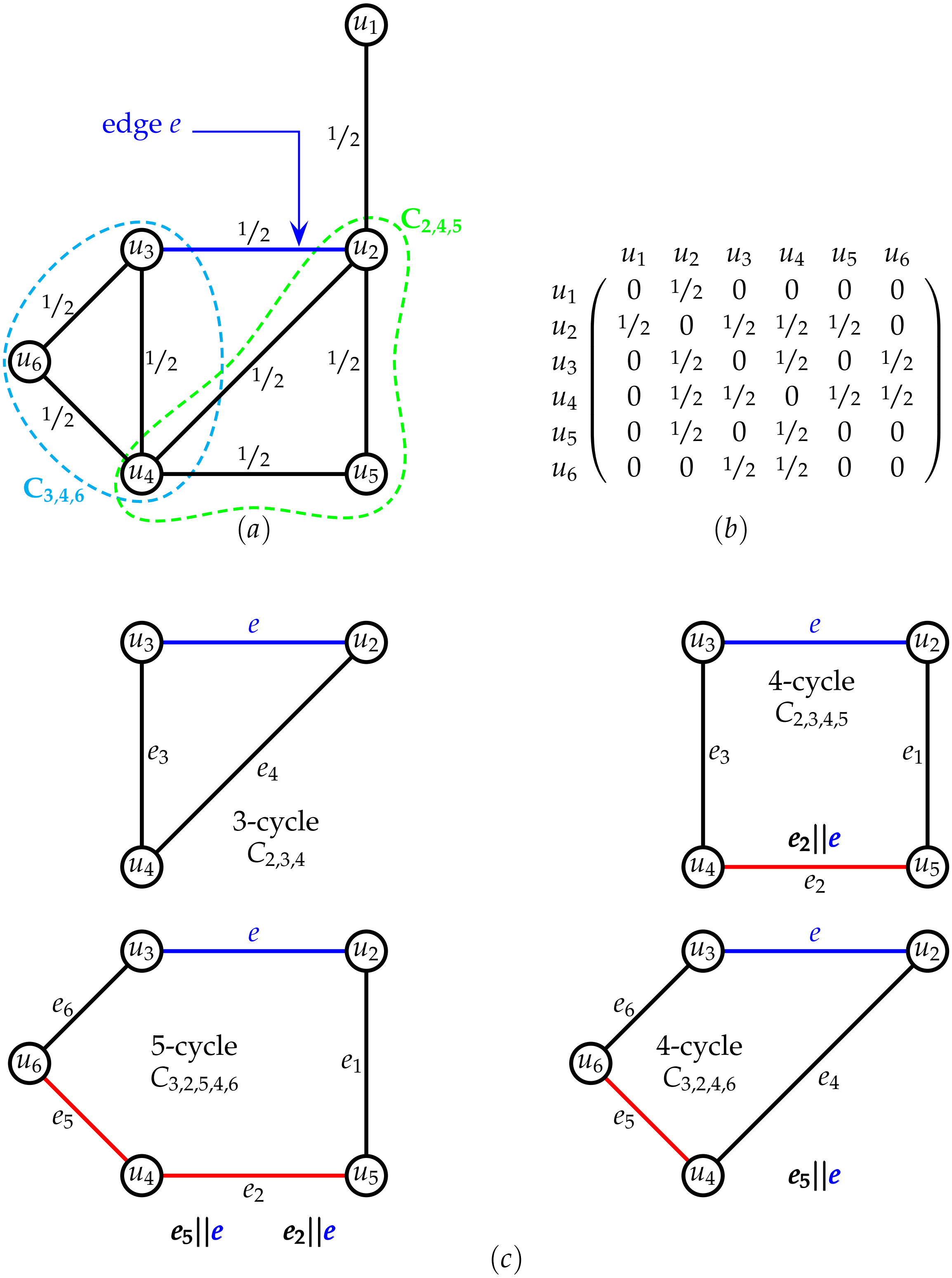

$$\begin{array}{c} \\ u_1 \\ u_2 \\ u_3 \\ u_4 \\ u_5 \\ u_6 \end{array}\begin{array}{c} \begin{array}{cccccc} u_1 & u_2 & u_3 & u_4 & u_5 & u_6 \end{array} \\ \begin{pmatrix} 0 & 1/2 & 0 & 0 & 0 & 0 \\ 1/2 & 0 & 1/2 & 1/2 & 1/2 & 0 \\ 0 & 1/2 & 0 & 1/2 & 0 & 1/2 \\ 0 & 1/2 & 1/2 & 0 & 1/2 & 1/2 \\ 0 & 1/2 & 0 & 1/2 & 0 & 0 \\ 0 & 0 & 1/2 & 1/2 & 0 & 0 \end{pmatrix} \end{array}$$

Figure S1. A simple example for illustration of calculation of $\mathfrak{C}^{2,5}(e)$. (a) The given graph and the edge e (all edge weights are $1/2$). (b) The connectivity matrix for the graph. (c) Listing of all f_d^2's (for $d \in \{3,4,5\}$) along with the relevant parallel edges used in the calculation of $\mathfrak{C}^{2,5}(e)$.

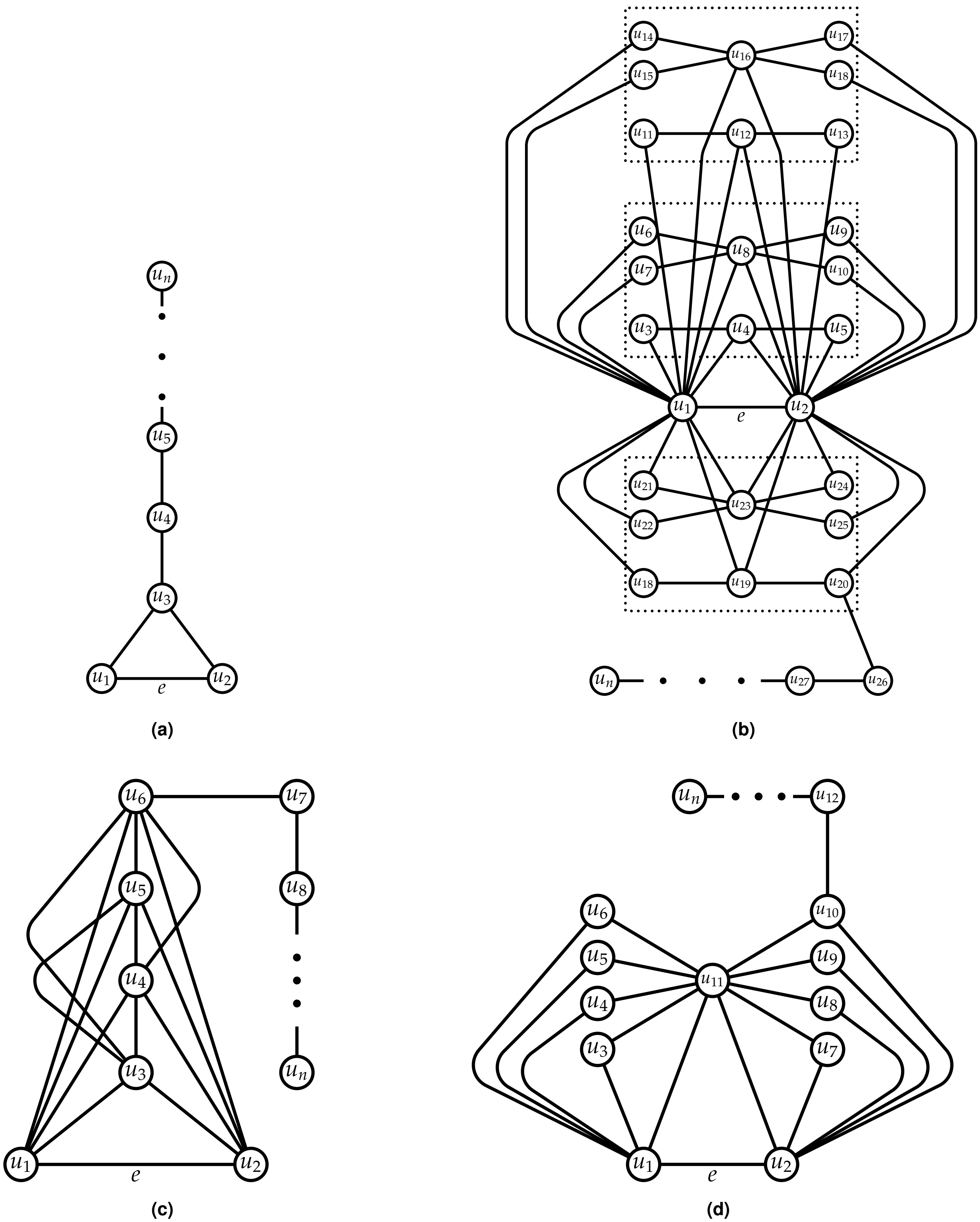

(a) (b) (c) (d)

Figure S2. **a.** A n-node graph for which $\mathrm{sgn}(\mathfrak{C}_G^{2,d}(e)) = \mathrm{sgn}(\mathfrak{C}_G^{\text{O-R}}(e)) = 1$. **b.** A n-node graph for which $\mathrm{sgn}(\mathfrak{C}_G^{2,d}(e)) = 1$ and $\mathrm{sgn}(\mathfrak{C}_G^{\text{O-R}}(e)) = -1$. **c.** A n-node graph for which $\mathrm{sgn}(\mathfrak{C}_G^{2,d}(e)) = -1$ and $\mathrm{sgn}(\mathfrak{C}_G^{\text{O-R}}(e)) = 1$. **d.** A n-node graph for which $\mathrm{sgn}(\mathfrak{C}_G^{2,d}(e)) = \mathrm{sgn}(\mathfrak{C}_G^{\text{O-R}}(e)) = -1$.
